# HSV-1 and Endogenous Retroviruses as Risk Factors in Demyelination

**DOI:** 10.3390/ijms22115738

**Published:** 2021-05-27

**Authors:** Raquel Bello-Morales, Sabina Andreu, Inés Ripa, José Antonio López-Guerrero

**Affiliations:** 1 Departamento de Biología Molecular, Universidad Autónoma de Madrid, Cantoblanco, 28049 Madrid, Spain; sandreu@cbm.csic.es (S.A.); ines.ripa@cbm.csic.es (I.R.); ja.lopez@uam.es (J.A.L.-G.); 2 Centro de Biología Molecular Severo Ochoa, CSIC-UAM, Cantoblanco, 28049 Madrid, Spain

**Keywords:** herpes simplex virus type 1, transposable elements, herpesviruses, endogenous retroviruses, demyelination, multiple sclerosis

## Abstract

Herpes simplex virus type 1 (HSV-1) is a neurotropic alphaherpesvirus that can infect the peripheral and central nervous systems, and it has been implicated in demyelinating and neurodegenerative processes. Transposable elements (TEs) are DNA sequences that can move from one genomic location to another. TEs have been linked to several diseases affecting the central nervous system (CNS), including multiple sclerosis (MS), a demyelinating disease of unknown etiology influenced by genetic and environmental factors. Exogenous viral transactivators may activate certain retrotransposons or class I TEs. In this context, several herpesviruses have been linked to MS, and one of them, HSV-1, might act as a risk factor by mediating processes such as molecular mimicry, remyelination, and activity of endogenous retroviruses (ERVs). Several herpesviruses have been involved in the regulation of human ERVs (HERVs), and HSV-1 in particular can modulate HERVs in cells involved in MS pathogenesis. This review exposes current knowledge about the relationship between HSV-1 and human ERVs, focusing on their contribution as a risk factor for MS.

## 1. Introduction

Herpes simplex virus type 1 (HSV-1) is a neurotropic human pathogen belonging to the *Alphaherpesvirinae* subfamily [1]. It is one of the most widespread human viral pathogens [2], and although humans are natural hosts, this virus can infect several species and numerous cell types in vitro [3]. Primary infection takes place in epithelial cells, where HSV-1 typically causes labial and oral lesions before spreading to the sensory neurons of the peripheral nervous system (PNS). From there, it travels retrogradely towards the trigeminal ganglia, where it establishes a latent infection [4]. However, HSV-1 may also establish latency in central structures such as the olfactory bulb, the brainstem, or the temporal cortex. HSV-1 may later reactivate, either spontaneously or in response to stimuli such as fever, immunosuppression, or exposure to ultraviolet light [1]. During reactivation, HSV-1 travels anterogradely along the axon, replicating in the dermatome innervated by the sensory neuron latently infected.

In addition to labial and oral lesions, HSV-1 may cause more serious pathologies such as encephalitis or keratoconjunctivitis, and studies in animals and human patients have suggested a link between HSV-1 and demyelinating processes [5]. Among these disorders, the most prevalent one is multiple sclerosis (MS), a neurodegenerative disease of the central nervous system (CNS) characterized by demyelination, inflammation, gliosis, and oligodendrocytic and axonal loss due to blood–brain barrier (BBB) disruption [6]. MS is typically multifocal and multiphasic (relapsing-remitting) and is recognized by multifocal demyelinating lesions in both the white and gray matter of the brain and spinal cord [6,7]. It is thought to be caused by infiltration of immune cells into the CNS, and it can be associated with axonal degeneration. In addition, BBB damage has been suggested as an essential step in MS progression [8], although it is not clear whether BBB impairment is a cause or rather a consequence of the disease [9]. MS is a multifactorial disease whose unknown etiology is probably influenced by a complex web of interactions between genetic and environmental factors [10,11,12]. However, several viruses may be involved in this demyelinating disorder [13,14] including HSV-1, which may act as a risk factor by mediating processes such as molecular mimicry, remyelination, or regulation of endogenous retroviruses (ERVs) [5].

ERVs are remnants of ancient retroviral germline infections that persist in the genomes of vertebrates [15,16]. These elements have been implicated in crucial physiological processes such as placentation [17], immunity [18,19], and development [20,21,22], and their dysregulation can lead to different pathologies [16,23,24,25,26]. Several studies have demonstrated a sound epidemiological relationship between MS and ERVs, which are up-regulated in the brains of MS patients [27,28,29,30,31,32]. Herpesviruses have also been associated with regulation of human ERVs (HERVs) [26,33,34], and HSV-1 in particular can modulate HERVs in cells involved in MS pathogenesis [5]. Transcription of HERVs genes may be stimulated by several herpesviruses [23] including HSV-1, HHV-6, and varicella-zoster virus (VZV) in lymphocytes from MS patients [35]; HSV-1 in neuronal or brain endothelial cell lines [36]; cytomegalovirus (CMV) in kidney transplant recipients [37]; HHV-6 in T cell leukemia cell lines [38]; and Epstein–Barr virus (EBV) in T cell lines [39] and in peripheral blood mononuclear cells (PBMCs) from MS patients and astrocytic cell lines [40]. Here, we focus on current knowledge about the relationship between HSV-1 and HERVs as a risk factor for MS.

## 2. Transposable Elements

Transposable elements (TEs), discovered by the Nobel Laureate Barbara McClintock and described for the first time in 1950 [41], are mobile DNA sequences that have the capacity to move around within genomes [42]. For decades, TEs were considered “junk DNA”, although some researchers such as Roy Britten, Eric Davidson, and McClintock herself, defended a relevant and active role for TEs in biology [43]. Currently, it is widely recognized that TEs exert a relevant influence on genome structure and function, and play a direct role in the generation of morphological innovations [44]. In addition, TEs are essential elements in the regulation of gene expression including chromatin modification, splicing, and translation [45].

**Figure 1:**
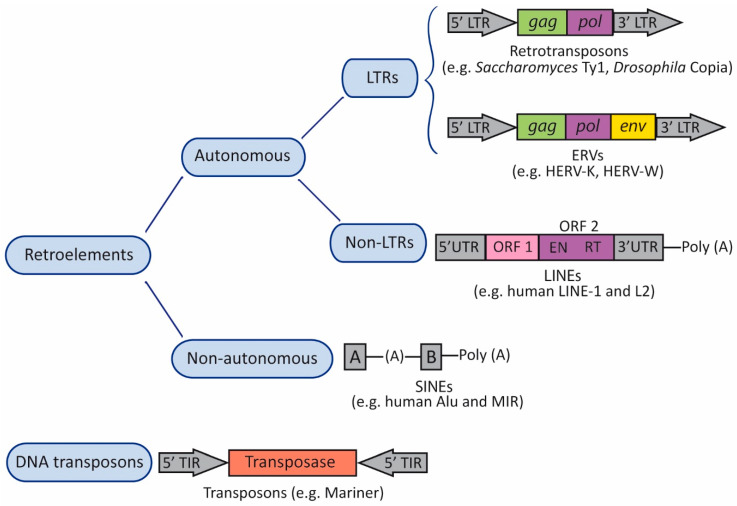
Transposable Elements (TEs). TEs can be organized into two major categories: retrotransposons (retroelements or class I elements) and DNA transposons (class II elements). Both types of TEs can be either autonomous or non-autonomous. Autonomous TEs encode reverse transcriptase (RT) and other proteins required for replication and transposition, whereas non-autonomous elements do not encode these proteins and need other TEs for their mobilization. Retrotransposons can be divided into two groups, according to the presence or absence of long terminal repeats (LTRs) flanking internal coding regions. The canonical autonomous LTR retrotransposons contain a small number of open reading frames (ORFs). Most elements contain an ORF including *gag* and *pol* domains, and endogenous retroviruses (ERVs) contain an ORF for *env*. *Gag* encodes a structural polyprotein, and *pol* encodes enzymatic activities: protease, RT, integrase, and ribonuclease H. ERVs contain a primer-binding site (PBS) located between the 5′LTR and *gag*, and a polypurine trait (PPT) located between *env* and the 3′LTR. The PBS binds the cellular tRNA priming the synthesis of the (–)strand DNA, and the PPT acts as a primer for the (+)strand DNA. Non-LTR retrotransposons include long interspersed nuclear elements (LINEs), and short interspersed nuclear elements (SINEs). The canonical LINE-1 element has two ORFs (ORF1 and ORF2) flanked by 5′ and 3′ UTRs; the 5′ UTR includes an RNA polymerase II promoter, and the element ends with a poly (A) tail. The canonical Alu element consists of two monomers (A and B) separated by an (A)-rich linker region, and ends with a poly (A) tail. A and B boxes are transcriptional promoters for RNA polymerase III. In DNA transposons, the transposase is flanked by terminal inverted repeats (TIRs).

TEs, which have been found across all three domains of life (bacteria, archaea, and eukarya), can be divided into two major categories: retrotransposons or class I elements; and DNA transposons or class II elements (Figure 1). Retrotransposons constitute the majority of the TEs present in the human genome [46]. Both retrotransposons and DNA transposons can be either autonomous or non-autonomous (Figure 1). Autonomous TEs encode reverse transcriptase (RT)—the enzyme that transcribes RNA back into DNA—and other proteins required for replication and transposition, and therefore do not need another element in order to move. On the contrary, non-autonomous elements do not encode these proteins and need other functional TEs for their mobilization [47]. Therefore, non-autonomous elements rely on an autonomous partner to provide the necessary proteins in trans [48]. In addition, retrotransposons can be divided into two groups, according to the presence or absence of long terminal repeats (LTRs) flanking internal coding regions (Figure 1). LTRs regulate expression, since they control the promoter activity and transcription of the retroelement [48]. LTR retrotransposons are abundant in animals and plants, whereas non-LTR elements are especially widespread in plant genomes. Non-LTR retrotransposons include long interspersed nuclear elements (LINEs) and short interspersed nuclear elements (SINEs) [49] (Figure 1). Retrotransposition of non-autonomous SINEs depend on proteins encoded by LINE-1 elements.

**Figure 2:**
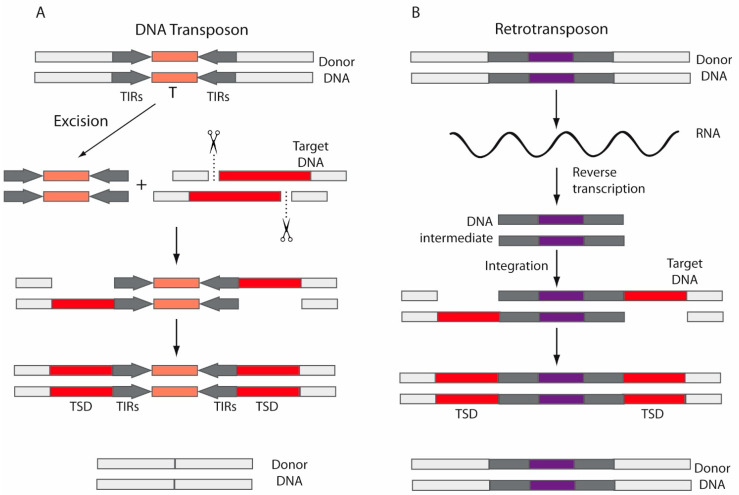
Mechanisms for mobilization. (**A**) DNA transposons move via a “cut and paste” mechanism, by which a transposase (T) mediates double-strand DNA cleavage and insertion. The DNA sequence is excised by the transposase from one region (donor DNA) and integrated into another region of the genome (target DNA). Transposases are flanked by terminal inverted repeats (TIRs). (**B**) Retrotransposons move via a “copy and paste” mechanism, using RT to transcribe the RNA back into DNA and integrases or endonucleases to insert it into a new location. After insertions, the DNA at the target site duplicates, producing target site duplications (TSDs).

DNA transposons are widespread across the three domains of life, although they are currently inactive in most mammals. These transposons move via a “cut and paste” mechanism (Figure 2A) by which a transposase mediates transposon movement by double-strand DNA cleavage and insertion. Thus, a DNA sequence is excised by the transposase from one region and integrated into another region of the genome [42,50]. Transposases are flanked by terminal inverted repeats (TIRs) (Figure 2A). On the contrary, retrotransposons move via a “copy and paste” mechanism (Figure 2B), using RT to transcribe the RNA back into DNA and an integrase or endonuclease to insert it into a new genomic location [51]. Therefore, unlike DNA transposons, retrotransposons are not replicated. When transposons are inserted into the new genomic location, the DNA at the target site duplicates, producing target site duplications (TSDs) (Figure 2A,B).

Almost half of the human genome is derived from TEs, with DNA transposons making up around 3% of the human genome [46]. Though this class of transposon is currently not mobile in the human genome, they were active during early primate evolution. On the other hand, LTR retrotransposons constitute around 8% of the human genome, whereas non-LTRs comprise about one-third of our DNA. The most abundant retroelements in mammals are the non-LTR retrotransposons of the LINE-1 family (L1), which alone comprise nearly 17% of the human genome [46].

Regarding integration, both retrotransposons and DNA transposons seem to insert non-randomly into the host genome and, generally, TEs integrate preferentially into specific domains of chromosomes where they can be less harmful [52]. LTR retrotransposons use RT to synthesize a double-stranded DNA (dsDNA) intermediate from the RNA template (Figure 2B). Then, that complementary DNA (cDNA) is inserted in the target DNA by an integrase; retroviruses also use integrases as a DNA insertion mechanism. On the contrary, non-LTR retrotransposons encode endonucleases, and copy their RNA directly into the target DNA via a target-primed reverse transcription mechanism [42,52].

Occasionally, transposition events can occur in the germline, leading to changes that pass on to the next generations. On the contrary, transposition events that take place in somatic cells can give rise to mosaicisms within individuals [53].

## 3. Transposable Elements, Exaptation, and Human Evolution

Although many TEs do not exert a relevant effect in the host genome, some insertions can be mutagenic, and therefore hosts have developed a variety of strategies to repress TE expression [54]. Although random mutation is an important source of TE inactivation, hosts have evolved other adaptive responses to impede retrotransposon proliferation, including transcriptional silencing through epigenetic modifications and post-transcriptional silencing through RNA interference [48,55]. In fact, only a small proportion of TEs remains actively mobile [56]. The non-LTR retrotransposon L1 and the SINEs Alu and SVA, as well as the LTR retrotransposons belonging to the HERV-K family, are the only currently active TEs in humans [46,57].

However, the host’s silencing mechanisms are not always effective, and the evolutionary success of TEs is undeniable. They are ubiquitous and account for a large fraction of eukaryotic genomes; they have driven many key evolutionary innovations and resulted in genetic diversity and evolutionary success [51,58]. They have even been suggested to play a possible role in speciation [59]. The evolutionary success of TEs may be due to several factors, including evasion from host epigenetic modifications and silencing. However, TEs have also evolved symbiotic relationships with the hosts that diminish their cost of propagation [60].

**Figure 3:**
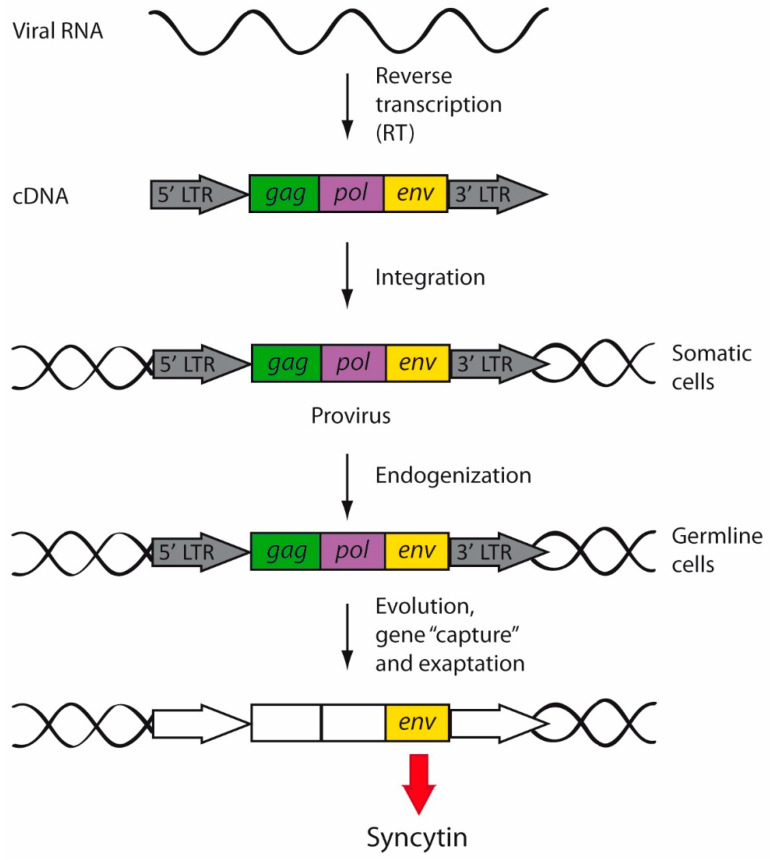
Retrovirus endogenization and exaptation. During replication, retroviral RNA is reverse-transcribed, giving rise to a double-stranded cDNA provirus that will be then integrated into the cellular genome of somatic cells. However, when the exogenous retroviruses infected germline cells, the integrated retroviruses began to be inherited in a Mendelian fashion. Endogenized retroviruses were vertically transmitted and fixed into the human genome. Over the course of evolution, endogenous retroviruses accumulated mutations (white boxes) and underwent gene capture and exaptation, by which retroviral genes started to perform new physiological functions. For example, syncytins are *env* genes of retroviral origin captured by mammals.

TEs are considered “selfish” genetic elements, since they exploit host cellular functions to increase in copy number and enhance their own transmission without a benefit to the host [61,62]. In fact, most TEs are neutral to the host, and they have been fixed through genetic drift. However, TEs can also be advantageous by generating evolutionary innovations [63]. A relevant mechanism by which TEs contribute to genome evolution is through exaptation [63,64] (Figure 3). This term was conceived and reported for the first time by Stephen J. Gould and Elisabeth Vrba in 1982 [65]. Exaptations are features that increase fitness in the present, but were not acquired by natural selection for their current role [65]. The classic example is the exaptation of feathers; the initial function of feathers in a flightless ancestor was insulation, but later in evolution, feathers were co-opted for flight in birds.

There are several well characterized exapted TEs, such as RAG genes in vertebrates, FHY3 transcription factors in plants, and several mammalian *env* genes (derived from ERVs) that are involved in placental development, including ERV-3, Peg10, or syncytins [63,66]. In addition, several TEs have been co-opted by prokaryotes and eukaryotes to be used as part of defense systems against infectious agents, including viruses and TEs themselves [67].

## 4. Transposable Elements and Human Disease

Once considered “junk” DNA, it is currently clear that TEs exert functional roles in physiological and pathological processes. TEs are important gene regulatory elements that act as alternative promoters, enhancers, or other elements [53]. In the human brain, TEs are usually silenced. However, dysregulation of those silencing mechanisms can lead to TE activation, giving rise to neurological disease [53,68]. In fact, it has been suggested that dysregulation of TEs might be involved in the etiology of neurodevelopmental and neurodegenerative disorders [69,70,71]. TEs have also been linked to cancer; a high level of somatic LINE-1 retrotransposition has been associated with epithelial tumors [55].

TEs can induce disease in different ways. First, although the genetic content of many TEs does not have a relevant effect on the host, sometimes insertions can disrupt genes. Second, the transcripts of TEs alone can be harmful, with TE-derived cytosolic nucleic acids leading to immune response. Organisms have developed several pathways for the sensing of intracellular nucleic acids (pattern recognition receptors [PRRs]), presumably in order to detect viruses within infected cells. While these pathways are crucial to trigger an effective antiviral response, unfortunately nucleic acid sensors may also be involved in several human autoimmune diseases [19]. Regarding TEs, the innate immune system may sense the cDNA, activating antiviral responses [19]. Thus, PRRs can detect TE-derived molecules, leading to nuclear activation of immune genes, which encode pro-inflammatory effectors such as cytokines and interferon (IFN) [53]. Third, some TE transcripts can also be translated into cytotoxic proteins. For instance, the HERV-K env protein contributes to neurotoxicity and neuronal death, and it has been suggested as a factor involved in the pathogenesis of amyotrophic lateral sclerosis (ALS) [72].

DNA methylation is the main strategy to silence TEs in higher eukaryotes, and genome expansion may be largely dependent on the action of DNA methyltransferases, which have evolved with TEs [73]. Typically, cancer cells display focal hypermethylation, often in 5′-cytosine-phosphate-guanine-3′ (CpG) islands, and global hypomethylation, particularly in repeated DNA sequences, retrotransposons, and endogenous retroviral elements [74,75]. Global DNA hypomethylation such as that seen in cancer [76,77] has also been associated with TE reactivation [78,79,80].

On the other hand, the brain can be considered to be a genomic mosaic, given the somatic mutations that appear during neurodevelopment. TEs are one source of somatic mosaicism, and interestingly, in mice, retrotransposition has been shown to be affected by experience, in particular by maternal care in the first weeks of life [81].

## 5. Human Endogenous Retroviruses (HERVs)

ERVs are vestiges of ancient retroviral infections that remain in the eukaryotic genomes. These TEs were acquired over thousands of years of evolution by the integration of retroviruses in the chromosomes of the host germline cells. Several HERVs [82,83], which collectively make up around 8% of the human genome, have been identified and characterized during the last decades [84,85,86,87]. HERV expression may be triggered by environmental factors. Although some HERVs may provide biological advantages, they also may induce pathogenesis in some circumstances; in fact, HERVs have been implicated in cancer and autoimmune diseases [72,88,89,90,91].

Exogenous retroviruses usually infect somatic cells and pass from one host to another by horizontal transmission. However, when certain ancestral exogenous retroviruses infected the germline, those proviral sequences were endogenized (Figure 3). From then on, the retroviral sequences started to be vertically transmitted to the offspring, being fixed in the whole population [16].

HERVs and exogenous retroviruses share the canonical proviral structure, composed of *gag*, *pol*, and *env* genes flanked by two LTRs. The retroviral *pol* gene encodes the enzymes protease, RT and integrase; the *gag* gene encodes the structural components matrix, capsid and nucleocapsid; and the *env* gene encodes the envelope surface and transmembrane proteins. However, during evolution, accumulation of mutations altered the structure of the majority of HERVs, which lost their coding capacity. Therefore, HERVs are inactive and cannot replicate, remaining only in a limited protein coding capacity or, more frequently, producing non-coding RNAs. Unlike mouse ERVs, no replication-competent HERVs have been described to date, although some maintain intact ORFs [92]. Thus, in contrast to the exogenous human retroviruses, HERVs are not infectious. In some cases, recombination between homologous LTRs resulted in the removal of the internal portion of DNA and giving rise to a solitary LTR. Exceptionally, HERV-K viruses can maintain a certain degree of activity and may transmit viral RNA to other cells [93].

The transcripts of HERVs are not pathogenic alone, and do not seem to exert relevant biological effects [23]. Furthermore, not even the proteins from HERVs have been shown to be pathogenic. A recombinant MS-associated retrovirus (MSRV) env protein, for instance, triggered an abnormal immune response in vitro, whereas, on the contrary, no significant effect was observed with the gag protein produced in the same system [94].

**Figure 4:**
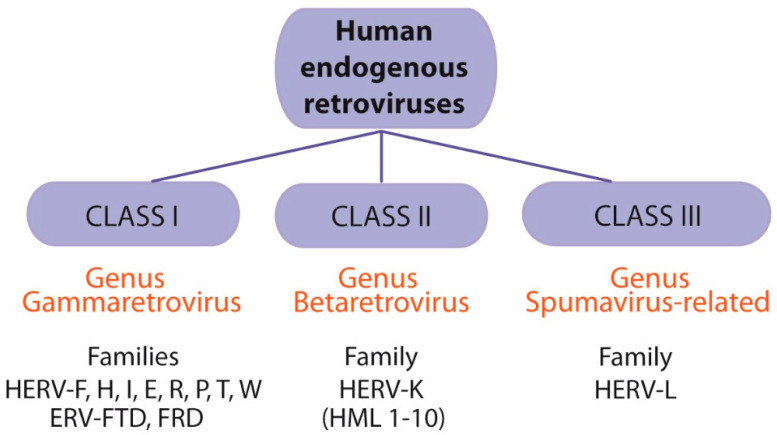
Classification of human endogenous retroviruses (HERVs). HERVs can be classified into three groups: class I (genus Gammaretrovirus), class II (genus Betaretrovirus), and class III (genus Spumavirus-related). Genus Gammaretrovirus includes families HERV-F, H, I, E, R, P, T, W as well as ERV-FTD and FRD. Genus Betaretrovirus includes the HERV-K family (HML1-10 subfamilies). Genus Spumavirus-related family includes the HERV-L family.

Several systems have been proposed to classify and name HERVs. A widely used nomenclature is based on the amino acid specificity of the tRNA that binds to the primer-binding site (PBS) to elicit reverse transcription. The one-letter code for the corresponding amino acid is added to the acronym HERV: HERV-H, -T, -W, -K, etc. [71,95]. On the other hand, an accepted classification is based on similarity to their exogenous counterparts. Thus, HERVs can be organized into three classes: class I (genus Gammaretrovirus), class II (genus Betaretrovirus), and class III (genus Spumavirus-related) (Figure 4). Families HERV-F, H, I, E, R, P, T, W as well as ERV-FTD and FRD belong to genus Gammaretrovirus. Betaretroviruses contain the HERV-K family (HML1-10 subfamilies), and the Spumavirus-related family includes the HERV-L family (Figure 4).

### 5.1. Endogenous Retroviruses and Multiple Sclerosis

HERVs expression has been linked to several diseases affecting the CNS [96], especially MS and ALS. Regarding ALS, several studies have associated this neurodegenerative disease with the HERV-K family [23,72], although recent reports have questioned this hypothesis [97], opening up an interesting field of debate [98,99]. Concerning MS, in 1998, Christensen and colleagues observed that PBMCs from the serum of patients with MS produced type C retrovirus-like particles, which were different from known retroviruses and had RT activity [100]. Later, the authors found an increased level of antibodies against HERV-H peptides in the serum and cerebrospinal fluid (CSF) of MS patients [101]. Those early observations suggesting activation of ERVs in MS patients supported these TEs as possible pathogenic factors for this disease [29,102]. Several HERV transcripts and proteins have been associated with neuroinflammation, which can activate HERVs through epigenetic dysregulation [23]. For instance, pro-inflammatory cytokines may up-regulate transcription of MSRV in cultured cells from MS patients [103]. In contrast, IFN-β therapy reduced the anti-env antibody reactivity for HERV-H and HERV-W [104] and, similarly, the MSRV load in the blood of MS patients decreased after one year of therapy with IFN-β [105]. MSRV may also induce human monocytes to produce major pro-inflammatory cytokines, and the increased IFN-γ, IL-6, and IL-12p40 found in PBMCs of MS patients correlated with disease severity in most cases [106,107].

It is established that HERVs are up-regulated in the brain of MS patients compared to healthy controls, and there is a strong epidemiological association between MS and the expression of HERVs [28,30,31,91,108,109,110]. The HERV-W family is a large group of TEs found in humans and also in non-human primates, and it is mobilized by the LINE-1 machinery [111]. Two retroviruses belonging to the HERV-W family have been proposed as major MS risk factors: MSRV and ERVWE1 [91].

#### 5.1.1. Multiple Sclerosis-Associated Retrovirus

MSRV is an important TE belonging to the HERV-W family [112] that has been linked to MS [27,30,31]. It has been proposed as a biomarker for MS behavior and therapeutic outcomes, supported by several facts. For instance, the presence of MSRV in the CSF of patients with optic neuritis (a disease that can precede the development of MS) can predict conversion to MS. MSRV in the CSF of patients at MS onset correlates with worst prognosis and disease progression. The genome of MS patients contains more MSRV DNA copies than in controls [91]. MSRV env expression has been observed in glial cells at the periphery of MS lesions and in astrocytes within the plaques [113]. Surprisingly, comparisons of peripheral blood between MS patients and healthy controls showed that MSRV expression is higher in an Eastern European population (with a lower risk of the disease) compared to the Northern European cohorts [114].

A more recent study demonstrated that the HERV-W env protein impaired oligodendroglial precursor cell (OPC) differentiation and remyelination. It mediated activation of Toll-like receptor 4 (TLR4) and induced pro-inflammatory cytokines and inducible nitric oxide synthase (iNOS), with a subsequent inhibition of oligodendroglial differentiation and decrease in myelin proteins [115]. In fact, MSRV env is a potent agonist of human TLR4 that induces TLR4-dependent pro-inflammatory stimulation of immune cells in vitro and in vivo, impairing OPC differentiation [116]. Another study tackled whether the pathogenic HERV-W env protein also plays a role in axonal damage in MS, finding that in MS lesions, the HERV-W env protein induced a degenerative phenotype in microglia, which then promoted damage to axons [117]. Besides the HERV-W env protein, HERV-H env expression is also increased in the B cells and monocytes of patients with active MS [118].

One early event during MS development is the compromise of the BBB, with major steps of pathogenesis being the adhesion of activated leukocytes to brain endothelial cells, and subsequent trans-endothelial migration through the impaired BBB [119]. In healthy individuals, brain-endothelial tight junctions limit adhesion and migration of immune cells into the CNS, but inflammation can increase expression of adhesion molecules such as intracellular adhesion molecule 1 (ICAM-1) and permit cells to cross the BBB. A recombinant MSRV env was able to stimulate expression of ICAM-1 and the pro-inflammatory interleukins IL-6 and IL-8 in an endothelial cell line [120]. Env protein was recognized via the TLR4 receptor, and treatment of brain endothelial cells with this MSRV protein significantly stimulated adhesion and trans-endothelial migration [120], demonstrating that MSRV can trigger TLR4-directed inflammation and increase BBB permeability.

Regarding prevention, management, and treatment of MS, research on HERV-W family can yield useful outcomes. For instance, Temelimab, or GNbAC1 antibody, is a monoclonal antibody that selectively binds to the HERV-W-Env and neutralizes it [121]. This drug is currently in clinical development for MS and type 1 diabetes mellitus, and phase 2 clinical trials have been completed with positive results.

#### 5.1.2. ERVWE1/Syncytin-1

**Figure 5:**
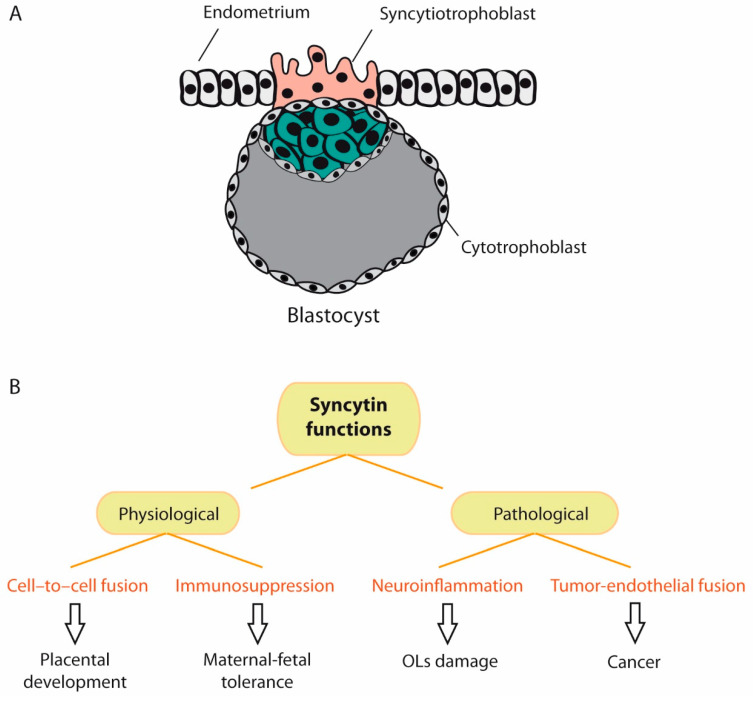
Physiological and pathological functions of syncytin. (**A**) When the embryo reaches the blastocyst stage, it undergoes implantation into the endometrium of the uterine wall. During implantation, the trophoblast (cells that form the outer layer of the blastocyst) develops into two layers: the cytotrophoblast and syncytiotrophoblast. The syncytiotrophoblast invades the maternal endometrium and directly contacts the maternal capillaries. Syncytin-1 plays a major role in syncytiotrophoblast cell fusion and, therefore, in embryonic development. (**B**). Besides cell-to-cell fusion, syncytin-1 exerts an immunosuppressive function that inhibits rejection of the fetus by the maternal immune system. However, syncytin-1 exerts also pathological functions, such as neuroinflammation and tumor-endothelial cell fusion. (OLs = oligodendrocytes).

Syncytin-1 is an env glycoprotein encoded by the replication-incompetent HERV-W element and is involved in mammalian placental morphogenesis [15,122]. It is encoded by a gene located on chromosome 7 (ERVWE1 locus), which contains a complete ORF, and it plays a crucial role in placental trophoblastic formation (Figure 5A). Syncytin-1 is involved in cell-to-cell fusion, and in addition, it exerts an immunosuppressive function that inhibits rejection of the fetus by the maternal immune system (Figure 5B). The process of fusion between cells and development of syncytia is similar to the process of fusion between viruses and cells during viral entry. Syncytin-1 can be found in eutherians and marsupials, all of which possess a placenta, and it has even been found in non-mammalian vertebrates [123].

A decrease in syncytin-1 expression and abnormal localization has been found in preeclampsia, a pregnancy disorder characterized by poor trophoblast differentiation and placental dysfunction [124]. On the other hand, syncytin-1 is up-regulated in glial cells of demyelinating lesions and in brain tissue of MS patients [109,125]. It is currently established that syncytin-1 can activate pro-inflammatory and autoimmune processes, triggering neuroimmune activation and oligodendrocyte injury [33,126] (Figure 5B). Syncytin-1 is also increased in breast cancer cell lines [127] and in endometrial carcinoma [128,129], and it is also an important mediator of tumor-endothelial cell fusion [130] (Figure 5B). The expression of syncytin-2, another fusogenic protein encoded by an HERV-FRD *env* gene [131], is also decreased in placentas from preeclamptic patients [132].

Syncytin-1 is different from MSRV env, although they share some sequence similarities [33,133]. Despite the fact that the *pol* sequences of MSRV and ERVWE1 share around 92% identity, HERV-W *env* genes are more heterogeneous [134]. MSRV env and syncytin-1 share several biological characteristics: both are potentially pathogenic, have pro-inflammatory and superantigenic properties, may trigger neurotoxicity, may cause neuroinflammation and neurodegeneration, and both have been proposed as risk factors for MS [91]. MSRV env and syncytin-1 are absent in healthy white matter, whereas they are up-regulated within acute and chronic MS lesions [134]. A significant difference between syncytin-1 and MSRV env is their localization: syncytin-1 is found inside the cell and on the plasma membrane, whereas MSRV can be visualized by electron microscopy as extracellular virus [134]. However, current tools do not permit easy discrimination between MSRV env and syncytin-1, and the origin of MSRV is not still clear; MSRV might be either an exogenous HERV-W, or a non-ubiquitous replication-competent member, or a partially defective but non-ubiquitous copy, occasionally complemented or recombined within the HERV-W retroviral family [40,91,135].

#### 5.1.3. HERV-H

In 2000, Christensen and colleagues [136] demonstrated a specific association between MS and the HERV-H family of retroviruses in cell cultures from MS patients. In a subsequent study, an increased immune response to HERV-H env correlated with disease activity [137]. However, a later study did not find HERV-H or HERV-W sequences in the CSF of MS patients [138]. Later reports suggested that HERV-H up-regulation in the lymphocytes of MS patients might induce anti-HERV antibodies or cell-mediated immune responses against gag and env peptides [33]. In 2002, Patzke and colleagues [85] identified and characterized a HERV gag transcript in a human pre-B cell leukemia cell line whose PBS was complementary to phenylalanine tRNA, common for the HERV-F family [139], although the overall genome sequence was related to the HERV-H family. Therefore, this retroviral sequence was named HERV-H/F [85]. HERV-Fc1, which belongs to the HERV-H/F subfamily, has been linked to MS [140]. Regarding genetic susceptibility, a single nucleotide polymorphism in the HERV-Fc1 locus on the X chromosome has been linked to an increased MS risk [30,141,142,143].

### 5.2. Herpesviruses and MS

Several studies have suggested herpesviruses as risk factors for MS pathogenesis and other demyelinating processes [5,13,14,144,145,146]. Epidemiological studies have found a correlation between VZV and MS [147], and VZV DNA isolated from the CSF and PBMCs of MS patients was increased during relapses compared to during remission and in healthy controls [148]. Oligoclonal bands (OCBs) directed against HHV-6 and EBV have been identified in MS patients [149], and OCBs against HSV-1 in the CSF of MS patients has also been reported [150], although this was not corroborated in all studies [151]. HSV-1 has been linked to demyelination in animal models [152,153,154,155,156,157,158,159] and in humans [160,161,162,163,164,165,166,167,168], and several studies have suggested other herpesviruses including HHV-6 [146,164,169,170,171,172,173,174], EBV [144,175,176,177,178,179,180,181] and HHV-8 [168,170,182] are risk factors for MS. In general, herpesvirus infections are more frequent in MS patients than in patients with other neurological diseases [138]. It has been postulated that EBV might initially activate HERV-W/MSRV, which, in turn, would trigger a future MS that would emerge years later [91]. HERV-W/MSRV has been proposed as a direct contributor to MS neuropathogenesis, both before and during the disease, or it might be a common link between several co-factors [91].

### 5.3. Herpesviruses, HERVs and MS

HERVs do not contain intact ORFs of essential retroviral genes, although several chromosomal copies may retain potential ORFs [183]. Hence, it has been proposed that exogenous viral transactivators, such as herpesviruses, might be key to reactivation of endogenous retroviral expression. Several studies have demonstrated that herpesviruses may activate HERVs, consistent with herpesviruses as risk factors for MS. Several studies have identified HSV-1, VZV, HHV-6, and EBV in MS patients, and it has been demonstrated that those viruses can trigger the expression of HERVs [184]. Transactivation of HERVs by exogenous viral infection might stimulate their expression in MS patients [184,185]. For example, EBV can transactivate the *env* gene of HERV-K18 in infected B cells [39], via the latent membrane proteins LMP-2A and LMP-1 [186]. HERV-K18 elicits superantigen activity, stimulating a large number of lymphocytes. EBV transactivates the HERV-K18 *env* gene through interaction with its entry receptor CD21 [187]. Therefore, a superantigen that was originally thought to be encoded by EBV itself was actually found to be a superantigen of HERV-K18 that was transactivated by EBV infection. The reactivation of endogenous viral superantigens by an unrelated herpesvirus, such as EBV or HSV-1, has been proposed as the “missing link” to explain the role of viral infection in the etiology of MS and other autoimmune diseases [188]. Apart from HERV-K18, the HERV-W family (which includes MSRV) exerts superantigen activity [188]. HHV-6 can also transactivate HERV-K18, either during latent or acute infection, through IFN-α produced by infected cells [38]. HSV-1 can also up-regulate the expression of HERV-W env protein in human neuroblastoma cell lines [188].

## 6. HSV-1 and HERVs: Implications for MS

### 6.1. HSV-1 and MSRV

**Figure 6:**
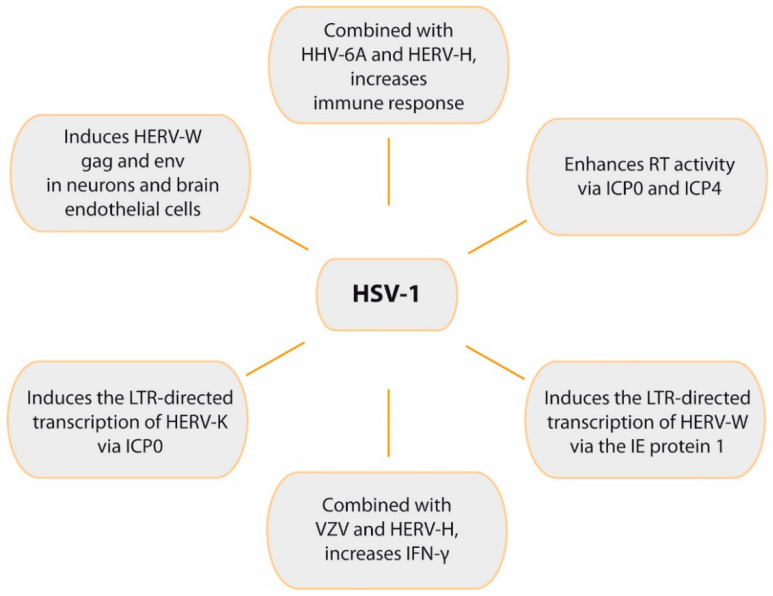
Role of HSV-1 in HERVs activation and immune response. The figure summarizes relevant effects of HSV-1 on HERVs transcription and the synergistic effects of both viruses on immune response.

Focusing on HSV-1, early studies showed that leptomeningeal cells from an MS patient expressed specific viral RT activity, whereas electron microscopy analysis revealed the presence of unidentified viral particles. RT activity was enhanced after viral transactivation by HSV-1 infection [189] (Figure 6). Similar findings were later obtained in monocyte cultures from MS patients [190]. The authors were able to transfer those unidentified viral particles, initially named LM7, to non-infected leptomeningeal cells in vitro [191]. After those first observations, HSV-1 infection of leptomeningeal cells from a MS patient demonstrated that the increased RT activity was mediated by ICP0 and ICP4 immediate early (IE) proteins, which strongly enhanced the expression of retrovirus-like particles harbored by the leptomeningeal cells [192]. These unknown retrovirus-like particles would be later identified as a novel HERV, belonging to the HERV-W family, and named MS-associated retrovirus, MSRV [193]. Later, a Danish group would also visualize retrovirus-like particles by electron microscopy of T cell cultures obtained from a patient with progressive MS [194]. To date, MSRV, which has been repeatedly isolated from MS patients, is the only HERV-W expressed as viral particles, and its association with MS has been later confirmed by several studies [195,196,197,198]. The presence and viral load of MSRV in blood and CSF of MS patients and healthy controls from different European regions were significantly associated with MS in all ethnic groups [102]. In addition, its presence in the CSF of MS patients has been related with a greater rate of disability and progression of the disease [33,199].

### 6.2. HSV-1 and ERVWE1/Syncytin-1

Using a HERV-W LTR reporter plasmid, early studies showed that HSV-1 infection can induce the LTR-directed transcription of HERV-W via the action of IE protein 1 (IE1) (Figure 6). This effect also required an Oct-1 binding site that is located in the LTR, suggesting that HSV-1 stimulates the LTR by increasing the DNA binding activity of Oct-1 transcription factor [200]. Syncytin-1 expression can be induced by viruses, such as HSV-1 or influenza, and cytokines such as TNF-α [134]. Research with MS patients demonstrated that syncytin-1 was up-regulated in MS lesions [125]. In addition, this env protein activated pro-inflammatory molecules in vitro (including IL-1β and iNOS), causing oligodendrocyte injury. This finding led to the hypothesis that syncytin-1 is involved in demyelination, mostly via cellular damage in the brain caused by redox reactants [125]. Subsequent in vitro studies found that HSV-1 can also induce HERV-W gag and env proteins in neurons and brain endothelial cells [36].

### 6.3. HSV-1 and HERV-K

HERV-K is the most recently acquired HERV family in humans (400,000–250,000 years ago) [33]. Unlike the majority of HERVs, the HERV-K family maintains intact ORFs for all retroviral genes [201]. HERV-K includes 11 subfamilies, HML-1 to HML-11. The K18 member of the HML-2 subfamily (HERV-K18) has found to be a risk factor for MS [202] and, as explained before, the HERV-K18 *env* gene can be transactivated by EBV in infected B cells [39]. It has been demonstrated that HSV-1 can induce the LTR-directed transcription of HERV-K (Figure 6), an effect mediated by the action of the IE protein ICP0 and that requires the AP-1 binding site on the HERV-K LTR. ICP0 up-regulated AP-1 activity, suggesting that this IE protein increased transcription of HERV-K via AP-1 site [203].

### 6.4. HSV-1 and HERV-H

It has been demonstrated that a simultaneous presence of HERVs and herpesvirus antigens has a strong effect on immune responses. Thus, combinations of inactivated herpesviruses (especially HHV-6A and HSV-1) and HERV-H antigens greatly increased immune responses in vitro in PBMCs from MS patients and healthy controls [204]. This increase was synergistic for HHV-6A, HSV-1, and VZV antigens combined with HERV-H, whereas there was no such effect with CMV. To investigate whether the in vitro findings were relevant in vivo, the authors analyzed the ability of herpes antigens to activate HERVs [35]. The results showed that HSV-1, HHV-6A, and VZV, but not CMV, induced endogenous RT activity [35] mediated by HERV-H activation. The experiments were performed with inactivated herpesviruses and thus were infection-independent, suggesting that activation of HERVs was directly due to viral proteins. Once activated, HERVs may promote several mechanisms, such as molecular mimicry, neurotoxicity, or up-regulation of immune mediators [35].

A later study analyzed the synergy between herpesvirus antigens and HERVs in the release of pro-inflammatory cytokines in PBMCs [205]. When combined with HSV-1 and VZV, HERV-H significantly increased IFN-γ, a pro-inflammatory cytokine which is recognized to exacerbate MS. However, HERV-H alone did not induce cytokine production or cell proliferation. HHV-6A also induced RT activity and proliferative responses [205].

## 7. Conclusions

Numerous studies have demonstrated that herpesviruses may activate HERVs. In addition, HERV expression has been linked to MS. This is in accordance with experimental and epidemiological studies which suggest herpesviruses as risk factors for MS. Thus, transactivation of HERVs by herpesvirus infections might stimulate their expression in MS patients, triggering demyelination or contributing to disease severity. Among herpesviruses, HSV-1 may play a role in demyelination mediated by HERVs, and in this regard, it has been shown to up-regulate expression of the HERV-W env protein. HSV-1 infection may also enhance MSRV RT activity, induce the LTR-directed transcription of HERV-W, and up-regulate syncytin-1, which may activate pro-inflammatory molecules causing oligodendrocyte injury. HSV-1 can also induce HERV-W gag and env proteins in neurons and brain endothelial cells, and it can induce the LTR-directed transcription of HERV-K. Finally, HSV-1, HHV-6A, and VZV, but not CMV, can induce HERV-H RT activity. When combined with HSV-1 and VZV, HERV-H significantly increased IFN-γ, a pro-inflammatory cytokine which may exacerbate MS. These data encourage further study of the role of HSV-1 as a risk factor for MS and other demyelinating processes.

## Data Availability

Not applicable.
